# Sex difference in flux of 27‐hydroxycholesterol into the brain

**DOI:** 10.1111/bph.15353

**Published:** 2021-02-04

**Authors:** Cristina Parrado‐Fernandez, Valerio Leoni, Ahmed Saeed, Patricia Rodriguez‐Rodriguez, Anna Sandebring‐Matton, Carmen M. Córdoba‐Beldad, Paula Bueno, Chaitanya Chakravarthi Gali, Ute Panzenboeck, Angel Cedazo‐Minguez, Ingemar Björkhem

**Affiliations:** ^1^ Department of Neurobiology, Care Sciences and Society Karolinska Institute Solna Sweden; ^2^ Discovery and Research AlzeCure Pharma AB Huddinge Sweden; ^3^ Laboratory of Clinical Chemistry, Hospital of Desio, ASST‐Monza and School of Medicine University of Milano Bicocca Monza Italy; ^4^ Department of Laboratory Medicine Karolinska Institute Huddinge Sweden; ^5^ Division of Immunology and Pathophysiology, Otto Loewi Research Center Medical University of Graz Graz Austria; ^6^ Rare & Neurologic Diseases Research Therapeutic Area, Neurodegeneration Research Sanofi Pharmaceuticals Paris France

**Keywords:** 27‐hydroxycholesterol, blood–brain barrier, CYP7B1, neurodegeneration, sex differences

## Abstract

**Background and Purpose:**

The cerebrospinal fluid (CSF)/plasma albumin ratio (QAlb) is believed to reflect the integrity of the blood–brain barrier (BBB). Recently, we reported that QAlb is lower in females. This may be important for uptake of neurotoxic 27‐hydroxycholesterol (27OH) by the brain in particular because plasma levels of 27OH are higher in males. We studied sex differences in the relation between CSF and plasma levels of 27OH and its major metabolite 7α‐hydroxy‐3‐oxo‐4‐cholestenoic acid (7HOCA) with QAlb. We tested the possibility of sex differences in the brain metabolism of 27OH and if its flux into the brain disrupted integrity of the BBB.

**Experimental Approach:**

We have examined our earlier studies looking for sex differences in CSF levels of oxysterols and their relation to QAlb. We utilized an in vitro model for the BBB with primary cultured brain endothelial cells to test if 27OH has a disruptive effect on this barrier. We measured mRNA and protein levels of CYP7B1 in autopsy brain samples.

**Key Results:**

The correlation between CSF levels of 27OH and QAlb was higher in males whereas, with 7HOCA, the correlation was higher in females. No significant sex difference in the expression of *CYP7B1* mRNA in brain autopsy samples. A correlation was found between plasma levels of 27OH and QAlb. No support was obtained for the hypothesis that plasma levels of 27OH have a disruptive effect on the BBB.

**Conclusions and Implications:**

The sex differences are discussed in relation to negative effects of 27OH on different brain functions.

**LINKED ARTICLES:**

This article is part of a themed issue on Oxysterols, Lifelong Health and Therapeutics. To view the other articles in this section visit http://onlinelibrary.wiley.com/doi/10.1111/bph.v178.16/issuetoc

**What is already known**

- Higher plasma levels of neurotoxic 27‐hydroxycholesterol in males than in females.
- Higher QAlb in males than in females consistent with a more permeable BBB in males.

**What does this study add**

- Significant sex differences in the relation between QAlb and 27‐hydroxycholesterol and 7α‐hydroxy‐3‐oxo‐4‐cholestenoic acid in CSF.
- No support for a direct disruptive effect of circulating 27‐hydroxycholesterol on the BBB.

1 **What is the clinical significance**

- We discuss whether the lower flux of 27‐hydroxycholesterol into the female brain may reduce neurodegeneration.

**Abbreviations**
24OH: 24*S*‐hydroxycholesterol
27OH: 27‐hydroxycholesterol
7HOCA: 7α‐hydroxy‐3‐oxo‐4‐cholestenoic acid
7βOH: 7β‐hydroxycholesterol
AD: Alzheimer's disease
BBB: blood–brain barrier
CSF: cerebrospinal fluid
CYP27: sterol 27‐hydroxylase
CYP7B1: cytochrome P450 family 7 subfamily B member 1
NINCDS‐ADRDA: National Institute of Neurological and Communicative Disorders and Stroke and the Alzheimer's Disease and Related Disorders Association
pBCEC: porcine brain capillary endothelial cells
TEER: transendothelial electrical resistance

## 1 INTRODUCTION

Hypercholesterolemia in midlife is regarded to be a risk factor for Alzheimer's disease (AD) in spite of the fact that lipoprotein‐bound cholesterol does not pass the blood–brain barrier (BBB) (Kivipelto et al., 2001). A major metabolite of cholesterol is 27‐hydroxycholesterol (27OH) and there is a strong correlation between levels of this oxysterol and cholesterol in the circulation. In contrast to cholesterol itself, 27OH can cross the BBB and there is a continuous uptake of this oxysterol into the brain (Heverin et al., 2005). Given the negative effects of 27OH under both in vitro conditions and in vivo experiments with different mouse models, it is possible that the negative effects of hypercholesterolemia are mediated by 27OH (Björkhem et al., 2009; Loera‐Valencia et al., 2019). The negative effects of 27OH include memory defects (Heverin et al., 2015; Ismail et al., 2017), reduced production of the ‘memory protein’ Arc in hippocampus (Ismail et al., 2017; Mateos et al., 2009), reduced uptake of glucose by the brain (Ismail et al., 2017), decrease in key synaptic protein levels (Ismail et al., 2017), decrease in dendritic spine density (Merino‐Serrais et al., 2019), worsening of amyloid pathologies (Famer et al., 2007; Marwarha et al., 2013) and effects on the renin–angiotensin system in the brain (Mateos et al., 2011). It can be assumed that high levels of cholesterol are associated with increased flux of 27OH across the BBB. This flux is dependent upon the permeability of the BBB and disruption of the BBB, in different disease conditions, leads to a higher influx of 27OH (Leoni et al., 2013).

The ratio between albumin in plasma and cerebrospinal fluid (CSF) is regarded to be a marker for the integrity of the BBB. Recently, we showed that there is significant and consistent sex difference in this ratio (20–30%) in patients and healthy subjects from birth up to 90 years old (Parrado‐Fernandez et al., 2018). In addition to this, there is a highly significant sex difference in the level of 27OH in the circulation, with levels about 30% higher in males than in females (Dzeletovic et al., 1995). Thus, it is possible that the female brain is less exposed to 27OH than the male brain.

In the present work, we have obtained additional support for the proposal that there are significant sex differences in the flux of 27OH and its final metabolite, 7α‐hydroxy‐3‐oxo‐4‐cholestenoic acid (7HOCA), in the human brain. We have also studied the possibility that the higher levels of 27OH in the male circulation may have a direct disruptive effect on the BBB. We speculate that the lower exposure of the female brain to 27OH may reduce the negative effect of hypercholesterolemia on neurodegeneration in this sex. This work was presented at the ENOR conference in Edinburgh in September 2019.

## 2 METHODS

### 2.1 Design of the studies and ethical aspects

All the different clinical studies are reported in detail in the articles referred to below and were re‐examined here, only with respect to sex differences. All the studies were approved by the ethical committee at Karolinska University Hospital Huddinge, and informed consent was obtained from all the subjects involved.

### 2.2 Patients and control subjects

1. Patients and control subjects with headache without objective findings, 128 females (age 46 ± 17 years) and 116 males (age 52 ± 18 years). Subjects included in previous studies by Leoni et al. (2004, 2005, 2006, 2013) and Shafaati et al. (2007).
2. Control subjects with headache but without objective findings (subgroup included in the above heterogeneous group), 79 females (age 79 ± 15 years) and 35 males age (44 ± 15 years). Subjects included in previous studies by Leoni et al. (2004, 2005, 2006, 2013) and Shafaati et al. (2007).
3. Patients and control subjects with a defect in the BBB (13 males and 13 females). A defective BBB function was defined as an increased CSF/serum albumin ratio (14–160, normally <10). Diagnoses in this group included Guillain‐Barre's disease, meningitis, encephalitis, polyneuropathy, pareses and miscellaneous. These subjects were included in the previous study by Saeed (Saeed, Floris, et al., 2014).
4. Patients with different degrees of neurodegeneration diagnosed according to DSM‐IV and National Institute of Neurological and Communicative Disorders and Stroke and the Alzheimer's Disease and Related Disorders Association (NINCDS‐ADRDA) criteria (28 males and 38 females). Subgroup of subjects included in previous studies conducted by Gil‐Bea et al. (2010).

### 2.3 Assay of oxysterols

24*S*‐Hydroxycholesterol (24OH) and 27‐hydroxycholesterol (27OH) in plasma and CSF were measured by isotope dilution mass spectrometry after hydrolysis as previously described (Leoni et al., 2013). Also 7HOCA was measured by isotope dilution mass spectrometry as described by Saeed, Floris, et al., (2014). Note that according to Fakheri and Javitt (2012), the preferred nomenclature for 27‐hydroxylation and 27‐hydroxycholesterol should be (25R)26‐hydroxylation and (25R)26‐hydroxycholesterol. Here, we prefer to use 27‐hydroxycholesterol (27OH) to be consistent with our previous publications.

### 2.4 Human brain samples

Fresh frozen human brain specimens of frontal cortex and hippocampus were obtained from Karolinska Institutet Brain Bank (Sweden) and the Harvard Brain Tissue Resource Center (MA, USA).

### 2.5 Relative quantification in real time

Total RNA was isolated using the RNeasy® mini kit (Qiagen) including DNase treatment (RNase‐Free DNase Set, Qiagen). RNA samples were run on agarose gels to confirm no genomic DNA contamination, and the purity was assessed by RNA 260/280 ratio. Total RNA (500 ng) was then reverse transcribed using High‐Capacity cDNA Reverse Transcription Kit (Applied Biosystems). To perform real‐time RT‐qPCR assays, we used the relative standard method supplied by Applied Biosystem (Applied Biosystems, 2004). Serial dilutions of a reverse transcription product (cDNA) from human cell line were carried out to obtain standards curves containing 100, 50, 5, 0.5 and 0.05 ng μl^−1^ and run by duplicate in the same PCR plate as the experimental samples. Relative quantities of mRNA were determined by comparing the threshold cycle (Ct) of each sample with that of a standard curve run on the same PCR plate. Thermocycling and fluorescence detection was performed according to the TaqMan® Gene Expression Assays Protocol using an ABI PRISM® 7000 Sequence Detection System with a total volume of 20 μl in each well containing 10 μl of PCR Master Mix (Applied Biosystems), 2 μl of cDNA (corresponding to 15 ng of total RNA), 1 μl of each TaqMan® Gene Expression Assays primer (Applied Biosystems) and 7 μl of molecular grade RNase‐free water (Dharmacon GE Lifesciences, B‐003000‐WB‐100). Relative quantities of target genes were adjusted to relative quantities of RPLPO and normalized to controls conditions (set at 100%). Values are reported as the mean ± standard error of the mean (SEM).

### 2.6 Western blotting

Human samples for western blot analysis were prepared from 100‐mg frozen human tissue. Tissue was homogenized using a pestle motor homogenizer (Sigma‐Aldrich) at +4°C in 1% TritonTM X‐100, 150 mM NaCl, 1 mM EDTA, 1 mM EGTA, 50 mM Tris, pH 7.5, supplemented with phosphatase and protease inhibitors (Sigma‐Aldrich). Then, homogenates were sonicated and centrifuged for 10 min at 3500 x g to remove cell debris. Protein content was measured using the Pierce BCA kit (Thermo Scientific) according to the manufacturer's instructions. Equal amounts of protein (50 μg) were prepared in 5× Laemmli sample buffer (10% SDS, 325 mM Tris‐Cl, pH 6.8, 20% glycerol, 500 mM DTT, 0.4% bromophenol blue) and loaded into 10% sodium dodecyl sulphate (SDS) polyacrylamide gels. Precision Plus Protein Dual Xtra Standard (Bio‐Rad) was used as molecular weight marker. The samples were then separated by electrophoresis (120 mV, 1 h 30 min) in a MiniProtean system (Bio‐Rad). Proteins were transferred to nitrocellulose membranes (GE Healthcare) (100 mV, 1 h) and blocked in 5% non‐fat milk for 1 h at room temperature. Afterwards, the membranes were incubated overnight at 4°C with the primary antibodies CYP7B1 (Ab77157, mouse mAB, isotype IgG2a, 1:1000, Abcam, UK) and GAPDH (1D4, mouse mAb, isotype IgG1, 1:2000, Enzo, Sweden). Then, membranes were washed with Tris‐buffered saline pH 7.4 (50 mM Tris‐Cl, 150 mM NaCl) containing 0.1% Tween to eliminate unbound antibody followed by incubations with secondary antibodies IRDye 800CW goat anti‐mouse IgG (1:10,000) [AB_2687825] and IRDye 680RD goat anti‐mouse IgG (1:10,000) [AB_2651128] (LI‐COR Biotechnology—GmbH, UK). Signal detection was performed using an Odyssey imaging system (for the fluorescent secondary antibodies). Quantification of the bands was performed with the ImageStudioLite app and calculated from the band intensity, minus the surrounding background. Results were expressed as percentages of the values obtained from the appropriate controls and normalized to GAPDH expression.

### 2.7 Isolation and culture of porcine brain capillary endothelial cells

Brains from freshly slaughtered pigs (about 6 months old) were obtained from the local slaughterhouse, and porcine brain capillary endothelial cells (pBCEC) were isolated as first described by Franke et al. (2000) with minor modifications (Kober et al., 2017). In brief, meninges and secretory areas were removed from hemispheres, and pBCEC were isolated from the remaining cerebral cortex by sequential enzymic digestion and centrifugation steps as described. pBCEC were plated on collagen‐coated (60 μg ml^−1^) 75‐cm^2^ culture flasks in M199 medium (containing 1% penicillin/streptomycin, 1% gentamicin, 1 mM l‐glutamine and 10% horse serum). After 24 h, cells were rinsed twice with PBS to remove cell debris and non‐adherent cells, and were grown in fresh M199 medium (containing 1% penicillin/streptomycin, 1 mM l‐glutamine and 10% horse serum) until confluent. All cell culture incubations were performed at 37°C, 95% humidity and 5% CO_2_.

### 2.8 Transwell experiments

To establish polarized pBCEC cultures (Kober et al., 2017), cells were trypsinized and plated onto collagen‐coated (120 μg ml^−1^) 12‐well transwell filter plates (0.4‐μm pore size, Corning) at a density of 40,000 cells cm^‐2^. Cells were grown for 2–3 days depending on the transendothelial electrical resistance (TEER; 50 Ω cm^−2^). The tightness of the transwell culture was assessed by measuring TEER using an EndOhm tissue resistance measurement chamber and EndOhm ohmmeter (World Precision Instruments, Florida). TEER of collagen‐coated, cell‐free filters were used as blanks. Tight junction formation was induced (overnight) by adding DMEM/Ham's F‐12 medium containing 550 nM hydrocortisone, 1% penicillin/streptomycin and 0.7 mM l‐glutamine. Establishment of intact tight junctions was indicated by TEER rising above 150 Ω cm^−2^. Agents known to increase BBB permeability, 27OH (0.055 and 0.5 μM), 24OH (0.5 μM), 7β‐hydroxycholesterol (7βOH; 0.5 μM) or LPS (5 μM), were used as positive controls and were added to apical media containing 1% BSA. TEER were measured at indicated time points for up to 24 h. Six independent experiments with the above additions and use of aliquots from the same cell culture were performed (Figure 5). In addition, two separate independent experiments with other cell cultures were performed with *n* = 3.

### 2.9 Data and statistical analysis

It should be emphasized that all the studies were retrospective and primarily designed to study the relation between levels of oxysterols in CSF and the circulation in different populations of patients, rather than to document sex differences. Because of this, the size of the groups of males and females were not equal and varied in the different studies. All the analyses were performed blinded with respect to the identity (including sex) of the subject investigated.

In accordance with the recommendations of BJP, no statistical evaluations were performed where the group size was < 5. All the patient data concerned with CSF and plasma levels showed a Gaussian distribution, as shown by the Kolmogorov–Smirnov analysis, and thus, parametric tests could be used. We used one‐way ANOVA followed by Tukey's test for multiple comparison and Student's t test for group comparisons and Pearson's test for correlation. The level of significance was set to *P* = .05. All the outliers were included in all material presented except for Figure 2. If the two female outliers were included, the coefficient of correlation increased from 0.90 to 0.98 (which should be compared with the correlation coefficient of 0.38 in males).

### 2.10 Materials

The following materials were supplied as follows: 27OH, 24OH and 7βOH were from Avanti Polar Lipids (Alabaster, Ala, USA) and LPS‐EK Ultrapure (tlrl‐peklps) by InvivoGen (San Diego CA, EEUU).

### 2.11 Nomenclature of targets and ligands

Key protein targets and ligands in this article are hyperlinked to corresponding entries in the IUPHAR/BPS Guide to PHARMACOLOGY (http://www.guidetopharmacology.org) and are permanently archived in the Concise Guide to PHARMACOLOGY 2019/20 (Alexander et al., 2019).

## 3 RESULTS

### 3.1 Measurements of QAlb and level of 27OH in CSF in different groups of patients and controls

In previous work, we measured QAlb and levels of 27OH in CSF in 128 females and 116 males with different neurological diseases and with headache without objective findings (Leoni et al., 2004, 2005, 2006, 2013; Shafaati et al., 2007). As expected (Parrado‐Fernandez et al., 2018), QAlb was significantly lower in females (7.8 ± 5.7; means ± SD) than in males (13.3 ± 12.3) in these populations. The levels of 27OH in CSF were slightly but significantly lower in females (1.6 ± 1.2 ng ml^−1^) than in males (2.0 ± 1.3 ng ml^−1^). There was a highly significant correlation between levels of 27OH in CSF and QAlb in both females (*R* = 0.46) and males (*R* = 0.59).

**FIGURE 1:**
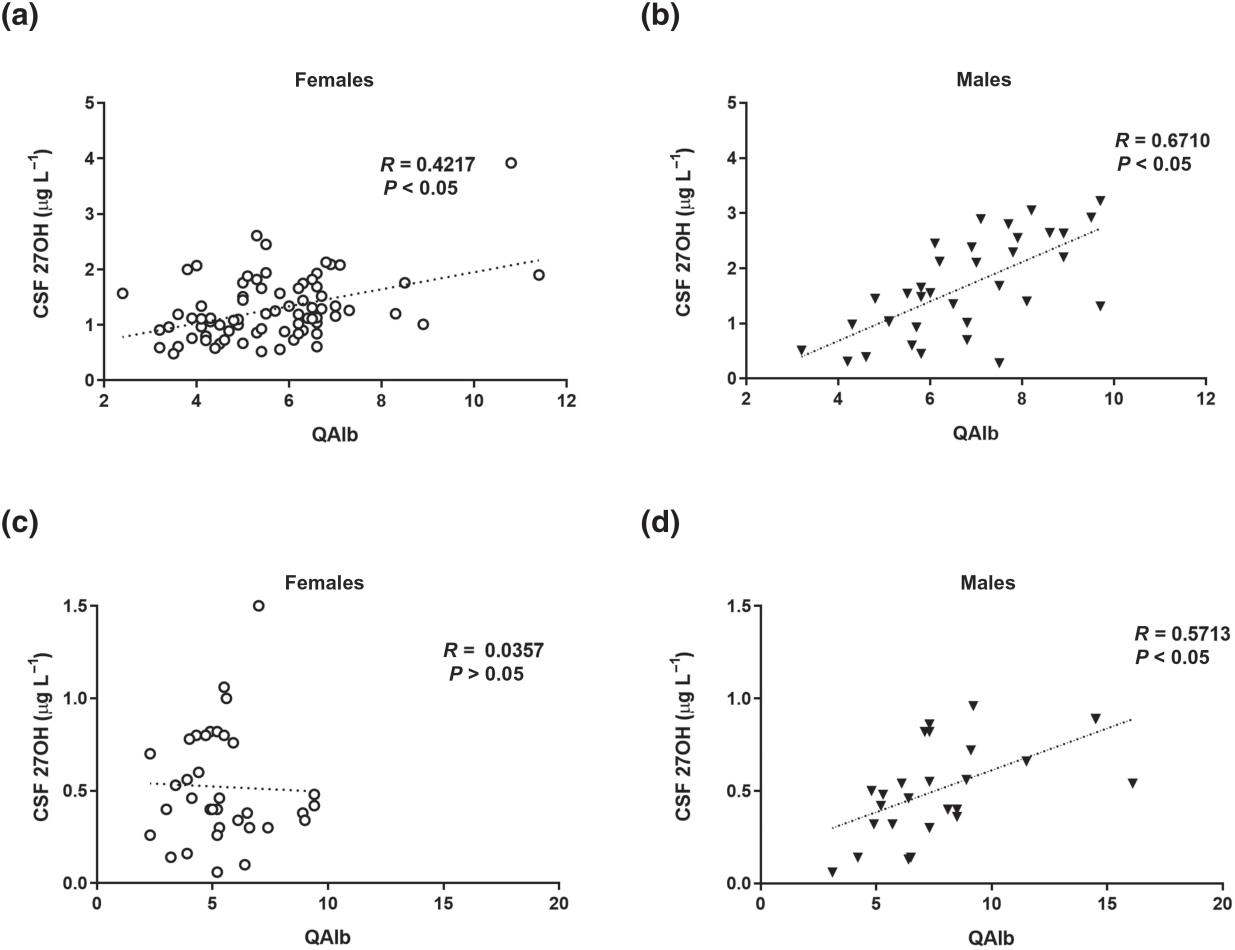
Correlation between cerebrospinal fluid (CSF)/serum albumin ratio and 27OH in CSF. Control subjects with headache but without objective findings, (a) 35 males and (b) 79 females. Patients with different degrees of neurodegeneration diagnosed according to DSM‐IV and NINCDS‐ADRDA criteria, (c) 28 males and (d) 38 females

The above populations are heterogeneous with several different diagnoses that could affect the integrity of the BBB by different mechanisms. Among the above subjects, 79 females and 35 males were regarded as controls (headache without neurological findings). In this homogenous subpopulation, there was a significantly lower QAlb in females (5.6 ± 1.6) than in males (6.7 ± 1.7). Also, in this subgroup, there was a highly significant correlation between levels of 27OH in CSF and QAlb in both females (*R* = 0.42) and males (*R* = 0.67) (Figure 1a,b). Altogether, in the above studies, the correlation between 27OH in CSF and QAlb was higher in males than in females.

In a separate study on a group of patients with different degrees of neurodegeneration, there was no significant correlation between 27OH in CSF and QAlb in females but a significant correlation in males (Figure 1c,d). In a separate group of patients with cognitive impairment, we observed that the correlation between QAlb and 27OH was lower (*R* = 0.43) in females compared with males (*R* = 0.87).

**FIGURE 2:**
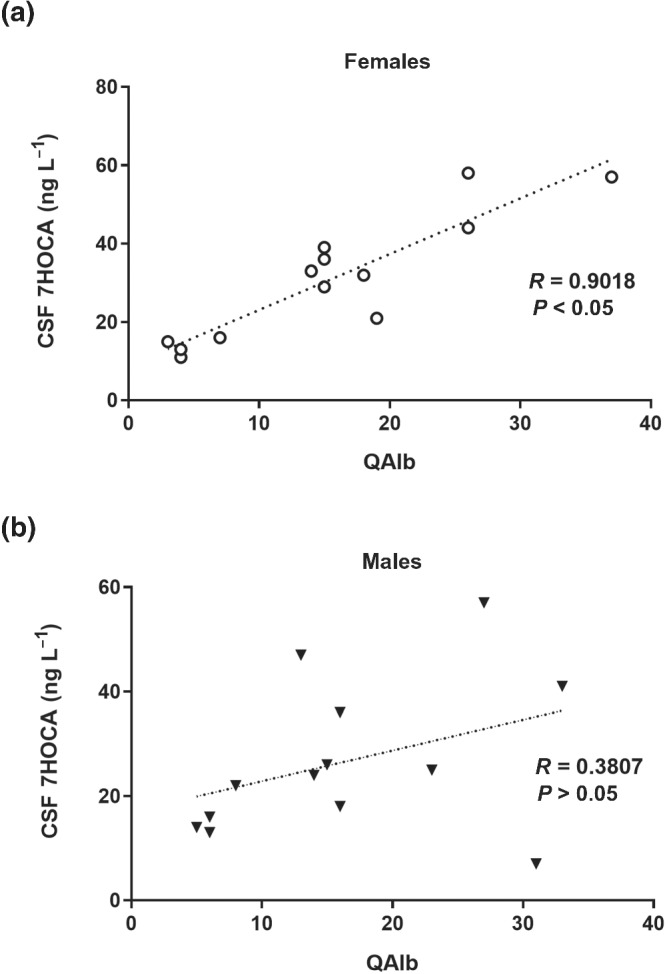
Correlation between cerebrospinal fluid (CSF)/serum albumin ratio and 7HOCA in CSF. Patients and control subjects with a defect in the blood–brain barrier (BBB), (a) 13 males and (b) 13 females

In the brain, the final metabolite of 27OH is 7HOCA(Saeed, Genove, et al., 2014). We have measured this metabolite in CSF from patients with different neurological diseases and found a significant correlation between the levels of this metabolite in CSF and QAlb (Saeed, Floris, et al., 2014). This correlation was highest in patients with a defective BBB. In a retrospective comparison between males and females in that study, we found that the degree of correlation between the two parameters was markedly different in the two sexes with a much higher correlation in females than in males (Figure 2a,b). There was no significant difference in the level of this metabolite in control subjects (10 males and 10 females, 11 ± 6 and 12 ± 3 ng ml^−1^ respectively; mean ± SD).

### 3.2 Measurements of QAlb and levels of 27OH in plasma

**FIGURE 3:**
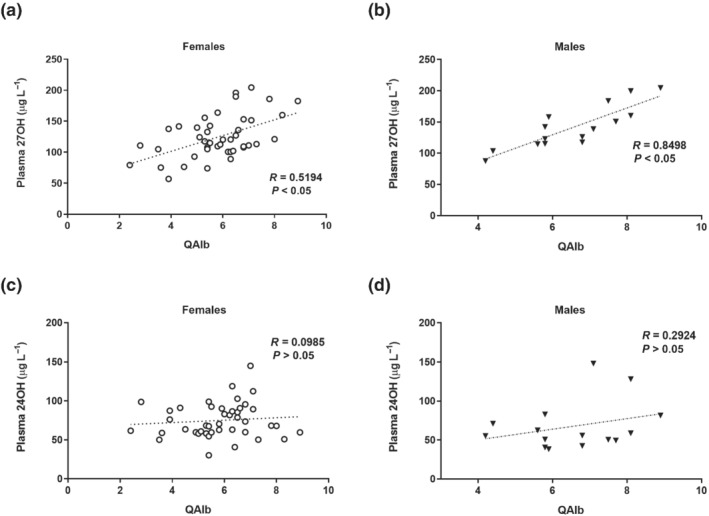
Correlation between cerebrospinal fluid (CSF)/serum albumin ratio and oxysterols in plasma. Control subjects with headache but without objective findings, 35 males and 48 females. (a,b) Correlation studies between plasma 27OH and QAlb. (c,d) Correlation studies between plasma 24OH and QAlb

We measured levels of 27OH in the circulation and compared these levels with the QAlb in patients with different degrees of neurodegeneration. A significant correlation was found (results not shown). This study was repeated with the control patients. As shown in Figure 3a,b, there was a significant correlation between the two parameters in both females (*R* = 0.52) and males (*R* = 0.85), with a higher correlation in males.

No significant correlation was observed between QAlb and plasma levels of 24OH (*R* = 0.1 in females and *R* = 0.29 in males) (Figure 3c,d). Also, there was no significant correlation between QAlb and plasma levels of cholesterol (*R* = 0.12 in females and *R* = 0.28 in males) (data not shown).

### 3.3 Measurements of CYP7B1 in hippocampus and frontal cortex of autopsy brain samples from males and females

**FIGURE 4:**
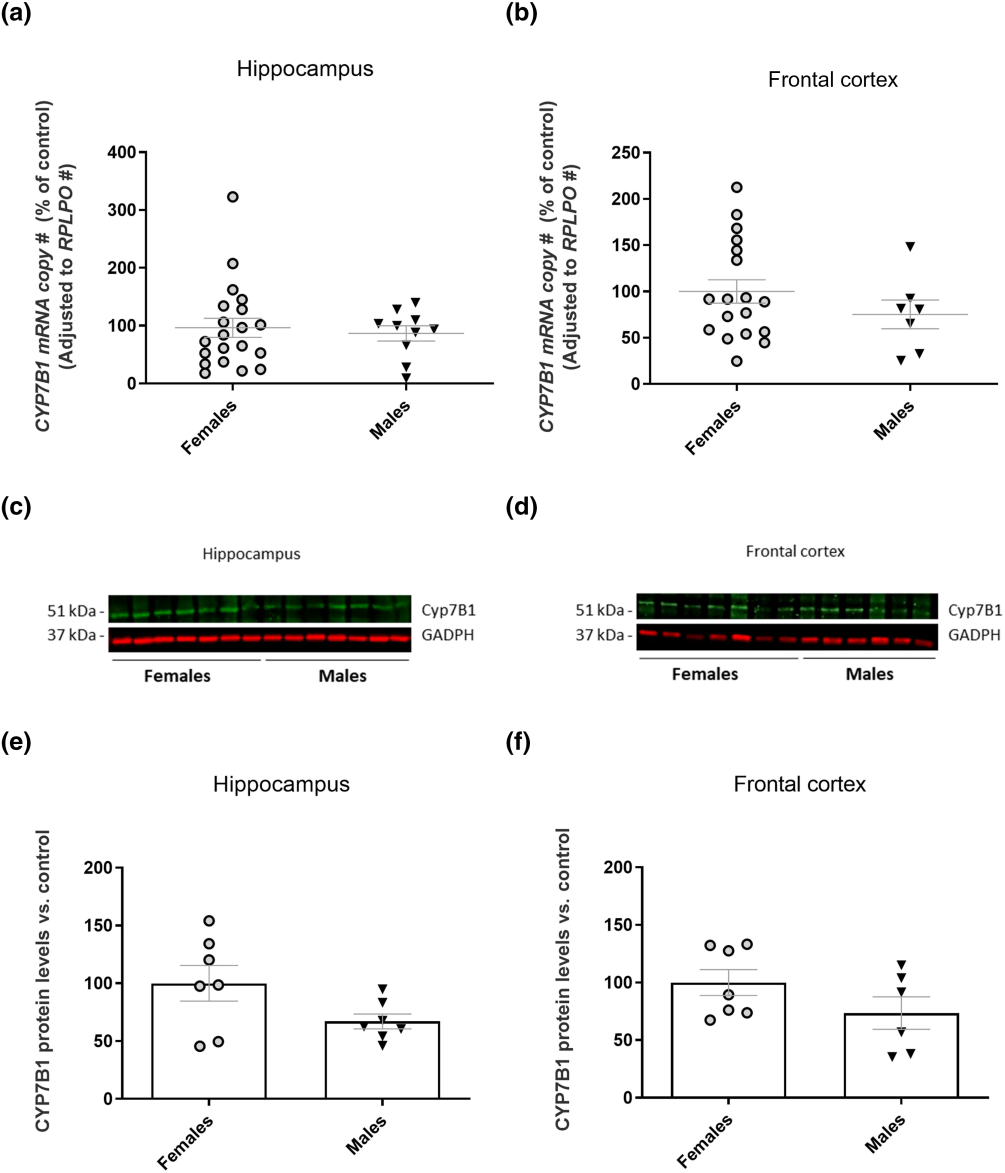
*CYP7B1* mRNA expression and protein levels in samples of human brain. Real‐time RT‐qPCR analysis of *CYP7B1* mRNA from (a) human hippocampus (females *n* = 23 and males *n* = 7) and (b) frontal cortex (females *n* = 18 and males *n* = 7). Representative western blot images of CYP7B1 from (c) human hippocampal homogenates (females *n* = 7 and males *n* = 7) and (d) frontal cortex homogenates (females *n* = 7 and males *n* = 6). (e,f) Respective histograms showing the quantification of the immunoreactivity measurements with data normalized against loading control (GAPDH) and expressed as a percentage of female group. Data are shown as mean ± SEM. Statistically significant differences were not detectable, using Student's *t* test

Relative mRNA and protein expression levels for *CYP7B1* in hippocampus and frontal cortex from control subjects showed no significant sex differences. As shown in Figure 4, when the study was repeated with samples from a heterogeneous group of subjects including both controls and patients with neurodegeneration, there was a tendency to higher mRNA and protein levels in females both in frontal cortex and hippocampus. This difference did not, however, reach significance.

### 3.4 Experiments with an in vitro model for the BBB

**FIGURE 5:**
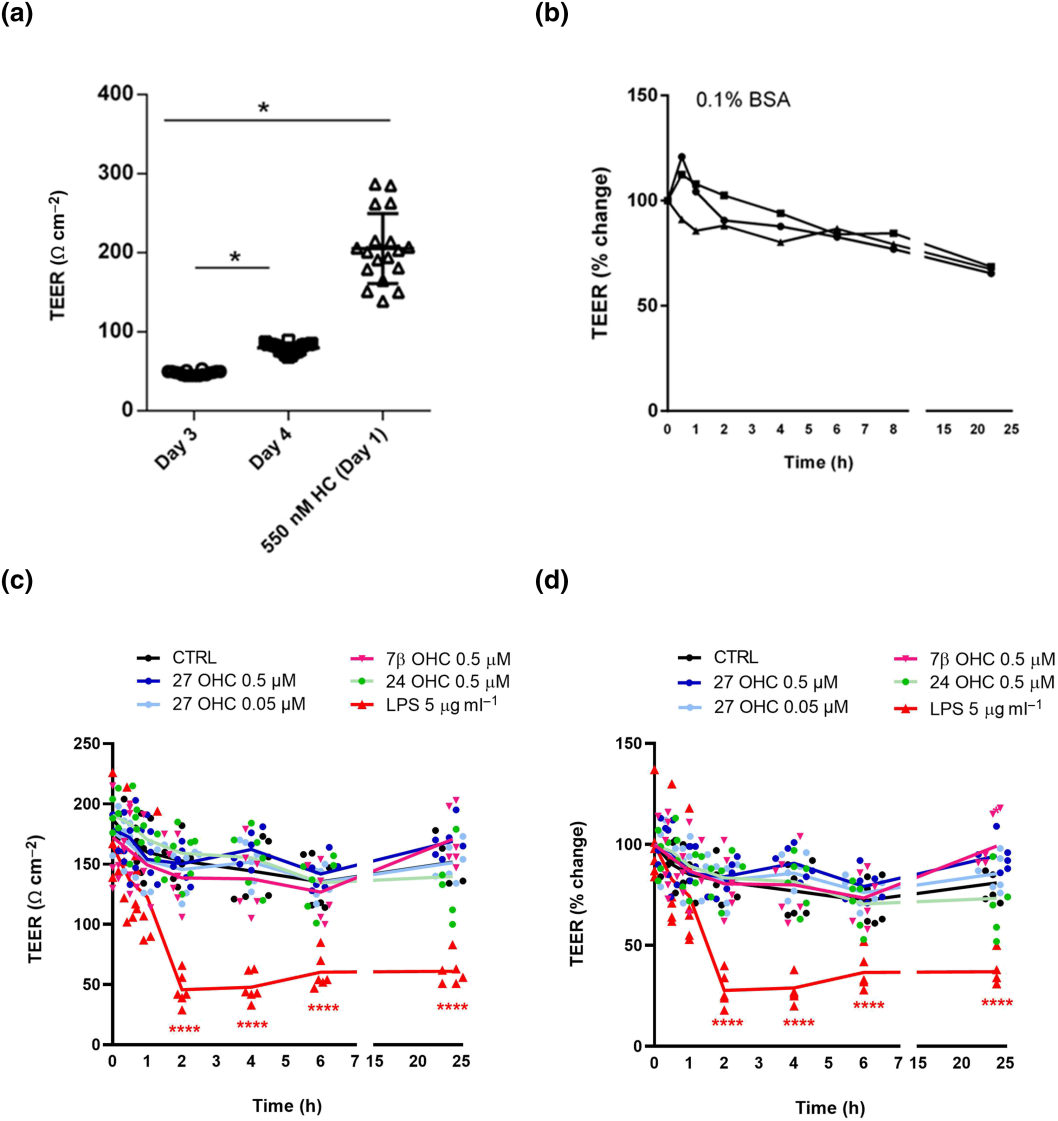
Effect of 24OH, 27OH, 7βOH and LPS on the blood–brain barrier (BBB) integrity. BBB permeability in vitro was assessed by measuring transendothelial electrical resistance (TEER) values of porcine brain capillary endothelial cells (pBCEC) grown on 12‐well transwell cell culture inserts. (a) TEER (Ω cm^−2^) was recorded on Day 3, Day 4 and a day after hydrocortisone‐mediated induction (550 nM) of tight junction formation. ^*^ *P* < .05, significantly different as indicated; two tailed, unpaired *t* test. (b) Percentage change in TEER values at 0, 0.5, 1, 2, 4, 6, 8 and 22 h after 0.1% bovine serum albumin (BSA) treatments. (c) TEER values (Ω cm^−2^) and (d) percentage change in TEER values at 0, 0.5, 1, 2, 4, 6, 8 and 22 h after vehicle (ethanol), 24OH (0.5 μM), 27OH (0.055 and 0.5 μM), 7βOH (0.5 μM) and LPS (0.5 μg ml^−1^) treatment. Data shown are means ± SD, (*n* = 6) from one experiment. The experiment was repeated twice with different cell cultures (total *n* = 3) showing the same pattern. * *P*< .05, significantly different from time = 0

Given the correlation between levels of 27OH in the circulation and QAlb, we tested the possibility that physiological levels of 27‐hydroxycholesterol may have a direct disruptive effect on the BBB. TEER values of brain endothelial cells growing on well cell culture inserts were recorded after hydrocortisone induction (Figure 5a) followed by registration of TEER changes after exposure to 0.1% BSA for different time intervals (Figure 5b). The TEER values were then recorded after exposure to 27OH (0.5 and 0.05 μM) during 25 h (Figure 5c,d). For reason of comparison, also 7βOH (0.5 μM) and 24OH (0.5 μM) were tested in the system. A positive control, LPS, (5 μg ml^−1^) was also included. The positive control had a significant effect on the permeability as evaluated by the change in TEER value (Figure 5c,d). There was, however, no significant effects of the different steroids on the TEER value. The experiment shown in Figure 5c,d with six replicates was repeated with different cell cultures with *n* = 3 showing the same pattern (results not shown).

## 4 DISCUSSION

As shown previously in healthy subjects and in anonymized patients, there is a clear sex difference in the degree of integrity of the BBB, as evaluated by measurement of the QAlb (Parrado‐Fernandez et al., 2018). Earlier, we had shown that a leaking BBB, due to pericyte deficiency, increases both influx and efflux of steroids across the BBB (Saeed, Genove, et al., 2014), implying that such loss of integrity affected flux of steroids in both directions. If both the influx of 27OH and the efflux of the metabolite 7HOCA are reduced in the brain of females, compared with those in males, there will be a tendency to retain both 27OH and 7HOCA in the brain of females. This may be the explanation for our surprising finding that, despite the lower influx of 27OH in the female brain, there was little or no difference in the levels of this oxysterol in CSF from males and females. A relative retention of the metabolite 7HOCA in the brain of females will be dependent upon the influx of 27OH and may explain the higher correlation between QAlb and CSF level of 7HOCA in females.

The rate of metabolism of 27OH in the brain may also be of importance. If this metabolism is higher in females than in males, it would tend to increase a relative accumulation of 7HOCA in the brain of females. The rate‐limiting step in the conversion of 27OH into 7HOCA is catalysed by *CYP7B1* (Meaney et al., 2007). The levels of *CYP7B1* mRNA levels in autopsy samples of brain were however similar in males and females.

**FIGURE 6:**
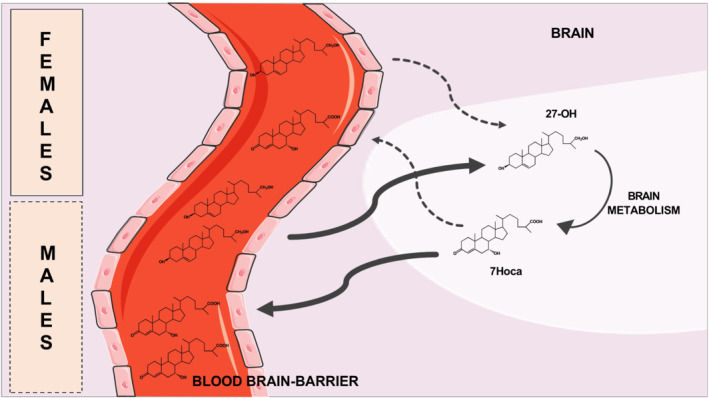
Schematic overview of the proposed mechanism behind the sex differences in the flux of 27‐hydroxycholesterol and 7HOCA in the brain. In males, higher influx of 27OH into the brain in relation to metabolism results in higher efflux of its metabolite 7HOCA In the female brain, the lower influx may be balanced by a lower efflux resulting in similar levels of oxysterols in the brain of the two sexes. Dotted arrows indicate lower flux

Figure 6 illustrates a possible mechanism behind the sex differences in the flux of 27OH and 7HOCA in the brain. The higher influx of 27OH in the male brain may be compensated for by a higher efflux of the final metabolite 7HOCA. In the female brain, the lower influx may be balanced by a lower efflux resulting in similar levels of oxysterols in the brain of the two sexes. We have previously found that the levels of 7HOCA in CSF from males and females (control subjects) are similar (Saeed, Floris, et al., 2014).

We found a significant correlation between 27OH in the circulation and QAlb (Figure 3). It is known that side chain oxidized oxysterols may affect the properties of biomembranes and increase their permeability (Bielska et al., 2014). In view of this, the possibility must be considered that 27OH in the circulation may affect the integrity of the BBB and that the lower integrity of the BBB in males may be related to this. In order to test this possibility, we used an in vitro model for the BBB with cultured brain endothelial cells. Physiological level of 27OH (0.5 μM) had no significant effect on the permeability of the barrier in this model. In another in vitro model using endothelial cells of another origin, a higher and unphysiological level of 27OH (10 μM) was shown to increase the permeability of the membrane (Dias et al., 2018). From our study, it may be concluded that physiological levels of 27OH in the circulation are not likely to affect the integrity of the BBB. Further support for this contention is obtained from patients with the hereditary spastic paresis of type SPG5 with a mutation in the *CYP7B1* gene. These patients with levels of 27OH up to 3 μM were recently reported to have normal QAlb (Schöls et al., 2017). We have used a mouse model with overexpressed CYP27 with high levels of 27OH in the circulation (Meir et al., 2002). If the high levels of 27OH cause a disruption of the BBB, higher levels of albumin would be expected in the brain of this transgenic mouse model. This was not found, however (unpublished observation). The data presented here in combination with the data from patients with a mutation in the *CYP27* gene and observations in mice with overexpression of CYP27 give strong support for the contention that 27OH in the circulation does not have a direct disruptive effect on the BBB.

At present, we have no explanation for the significant correlation between QAlb and levels of 27OH in the circulation. The possibility must be considered that there is an unknown circulatory factor with a level correlated to level of 27OH which has a regulatory effect on the permeability of the BBB. According to our studies, this regulatory factor cannot be identical to cholesterol or 24OH. If such a factor exists, its link to 27OH may be different in patients with SPG5.

We do not know if the negative effects of 27OH on the brain are related to the magnitude of the flux of the oxysterol into the brain or the actual concentration of it in the brain. The flux of the oxysterol into the brain is higher in males than in females whereas the concentration of the oxysterol appears to be similar in the brain of the two sexes. If the magnitude of the flux is most important and given the close relation between 27OH and cholesterol in the circulation, hypercholesterolemia would be expected to be associated with more negative effects in males than in females. In a recent Chinese cross‐sectional study, the relation between serum lipids and cognitive function in 1762 participants was studied (Zhao et al., 2019) High total cholesterol levels in the circulation were positively associated with cognitive impairment in elderly males but not in females. In a French study on 2737 men and 4118 women, a hypercholesterolemic pattern with high total cholesterol was associated with increased risk of cognitive decline in men but not in women (Ancelin et al., 2014). These studies agree with the hypothesis that hypercholesterolemia is a more serious risk factor for neurodegeneration in males than in females. More studies in different populations are required, however, before definitive conclusions can be drawn.

## AUTHOR CONTRIBUTIONS

I.B., A.C., C.P. and V.L. contributed to the conception and design of the work. V.L., C.P. and A.S. analysed the CSF and plasma. C.P., A.M., P.R., A.M., C.B. and P.B. performed the in vitro measurements. C.G. and U.P. performed the transwell experiments. I.B., A.C., C.P. and V.L. drafted the manuscript.

## CONFLICT OF INTEREST

The authors declare no conflict of interest.

## DECLARATION OF TRANSPARENCY AND SCIENTIFIC RIGOUR

This Declaration acknowledges that this paper adheres to the principles for transparent reporting and scientific rigour of preclinical research as stated in the BJP guidelines for Design & Analysis, and Immunoblotting and Immunochemistry, and as recommended by funding agencies, publishers and other organisations engaged with supporting research.

## Data Availability

The data that support the findings of this study are available from the corresponding author upon reasonable request. Some data may not be made available because of privacy or ethical restrictions.
